# Tumour regression and ERCC1 nuclear protein expression predict clinical outcome in patients with gastro-oesophageal cancer treated with neoadjuvant chemotherapy

**DOI:** 10.1038/sj.bjc.6605686

**Published:** 2010-05-11

**Authors:** K R Fareed, A Al-Attar, I N Soomro, P V Kaye, J Patel, D N Lobo, S L Parsons, S Madhusudan

**Affiliations:** 1 Laboratory of Molecular Oncology, Academic Unit of Oncology, School of Molecular Medical Sciences, Faculty of Medicine and Health Sciences, University of Nottingham, Nottingham, UK; 2 Department of Pathology, Nottingham University Hospitals, Nottingham, UK; 3 Division of Gastrointestinal Surgery, Nottingham Digestive Diseases Centre, NIHR Biomedical Research Unit, Nottingham University Hospitals, Nottingham, UK; 4 Department of Surgery, Nottingham University Hospitals, Nottingham, UK

**Keywords:** tumour regression grade, gastro-oesophageal cancers, neoadjuvant chemotherapy, ERCC1, APE1, p53

## Abstract

**Aims:**

Neoadjuvant chemotherapy followed by surgery is the standard of care for patients with gastro-oesophageal adenocarcinoma. Previously, we validated the utility of the tumour regression grade (TRG) as a histopathological marker of tumour downstaging in patients receiving platinum-based neoadjuvant chemotherapy. In this study we profiled key DNA repair and damage signalling factors and correlated them with clinicopathological outcomes, including TRG response.

**Methods and results:**

Formalin-fixed human gastro-oesophageal cancers were constructed into tissue microarrays (TMAs). The first set consisted of 142 gastric/gastro-oesophageal cancer cases not exposed to neoadjuvant chemotherapy and the second set consisted of 103 gastric/gastro-oesophageal cancer cases exposed to preoperative platinum-based chemotherapy. Expressions of ERCC1, XPF, FANCD2, APE1 and p53 were investigated using immunohistochemistry.

In patients who received neoadjuvant chemotherapy, favourable TRG response (TRG 1, 2 or 3) was associated with improvement in disease-specific survival (*P*=0.038). ERCC1 nuclear expression correlated with lack of histopathological response (TRG 4 or 5) to neoadjuvant chemotherapy (*P*=0.006) and was associated with poor disease-specific (*P*=0.020) and overall survival (*P*=0.040).

**Conclusions:**

We provide evidence that tumour regression and ERCC1 nuclear protein expression evaluated by immunohistochemistry are promising predictive markers in gastro-oesophageal cancer patients receiving neoadjuvant platinum-based chemotherapy.

Neoadjuvant platinum-based chemotherapy followed by surgery is the standard of care for patients with gastro-oesophageal adenocarcinoma (Medical Research Council Oesophageal Cancer Working Group, 2002; Cunningham *et al*, 2006). We recently reported the utility of tumour regression grade (TRG) analyses as a marker of histopathological response and tumour downstaging in tumours receiving neoadjuvant chemotherapy (Fareed *et al*, 2009). TRG was defined as per Mandard's criteria (Mandard *et al*, 1994). In brief, TRG1 (complete regression) showed absence of residual cancer and fibrosis extending through the different layers of the oesophageal wall; TRG2 was characterised by the presence of rare residual cancer cells scattered through the fibrosis; TRG3 was characterised by an increase in the number of residual cancer cells but fibrosis predominated; TRG4 showed residual cancer outgrowing fibrosis; and TRG5 was characterised by the absence of regressive changes (Mandard *et al*, 1994). In the neoadjuvant chemotherapy (CS) group (*n*=84), 46.7% of gastric/gastro-oesophageal junction adenocarcinomas, and 45.5% of lower third oesophageal adenocarcinomas, had TRG 1, 2 or 3 compared with 13.7% in the primary surgery group (*n*=124; *P*<0.001 and *P*=0.006, respectively). In CS group, responders (TRG 1, 2 or 3) showed significant tumour downstaging (early ypT-stage disease (*P*=0.002)). In gastric cancers specifically, additional associations were observed with negative nodal disease (*P*=0.044) and absence of vascular invasion (*P*=0.027; Fareed *et al*, 2009).

The response rate to platinum-based chemotherapy in gastro-oesophageal tumours is approximately 40% (Medical Research Council Oesophageal Cancer Working Group, 2002; Cunningham *et al*, 2006, 2008). Although platinum therapy has been a major advance in improving patient outcomes, the development of treatment-related toxicity and the emergence of resistance (both intrinsic and acquired) limit the effectiveness of platinating agents in solid tumours (Siddik, 2003; Rabik and Dolan, 2007). Platinum interacts with DNA to form predominantly intra-strand crosslink DNA adducts that trigger a series of intracellular events that ultimately result in cell death (Siddik, 2003; Zorbas and Keppler, 2005; Cepeda *et al*, 2007). DNA intra-strand crosslinks are processed and repaired by the nucleotide excision repair (NER) pathway in mammalian cells (Madhusudan and Hickson, 2005; Madhusudan and Middleton, 2005; Reardon and Sancar, 2005; Gillet and Scharer, 2006; Gossage and Madhusudan, 2007). Of the several factors involved, ERCC1-XPF heterodimer has been shown to have an important role in NER. Inactivation of NER results in platinum hypersensitivity in preclinical studies (Gossage and Madhusudan, 2007). In addition to NER, the Fanconi anaemia (FA) pathway has recently emerged as being critically involved in the regulation of DNA crosslink repair in mammalian cells (Levitus *et al*, 2006; Wang, 2007). Although the exact molecular mechanism is not completely known, it is clear that in response to crosslinkers, a complex consisting of at least eight FA proteins (A, B, C, E, F, G, L and M) monoubiquinates FANCD2, which is subsequently targeted to the chromatin in which it interacts with FANCD1 (also known as BRCA2) to facilitate DNA repair that may require the components of the nucleotide excision repair, homologous recombination and non-homologous end joining machinery (Mirchandani and D’Andrea, 2006; Wang, 2007). Disruption of the FA/BRCA pathway by germline mutations in *FA* genes results in crosslinker hypersensitivity (D’Andrea and Grompe, 2003; Chen *et al*, 2005; van der Heijden *et al*, 2005; Bartz *et al*, 2006; Wang *et al*, 2006; Zhang *et al*, 2006). Human apurinic/apyrimidinic endonuclease (APE1) is a multifunctional protein that has an essential role in DNA base excision repair involved in the repair of base damage, including oxidative base damages induced by platinating agents (Demple and Sung, 2005). Overexpression of APE1 results in relative resistance to platinating agents in preclinical models (Wang *et al*, 2009; Zhang *et al*, 2009). The p53 tumour suppressor protein has an essential role in DNA damage signal recognition and response. p53 is a transcription factor that binds to specific sites in the regulatory regions of p53-responsive genes, leading to cellular events such as growth arrest (perhaps to allow for DNA repair), or where there is extensive DNA damage to initiate apoptosis. p53 expression is common in gastro-oesophageal cancer and may influence response to chemotherapy (Kamoshida *et al*, 2007).

In this study we profiled key DNA repair and damage signalling factors involved in processing platinum-induced DNA lesions (ERCC1, XPF, APE1, FANCD2 and p53) and correlated them with clinicopathological outcomes in gastro-oesophageal cancer patients treated with platinum-based chemotherapy.

## Materials and methods

### Study design and setting

Investigation of the expression of DNA repair and DNA damage signalling factors in gastro-oesophageal cancers was carried out on two tissue microarray (TMA) sets. The first set consisted of 142 gastric/gastro-oesophageal cancer cases not exposed to neoadjuvant chemotherapy. With recent incorporation of neoadjuvant chemotherapy as a standard treatment option for operable gastro-oesophageal tumours (Cunningham *et al*, 2006), we also established a second TMA of 103 gastric/gastro-oesophageal cancer cases exposed to preoperative platinum-based chemotherapy. Tissue was obtained from patients treated at Nottingham University Hospitals (NUH) between 2001 and 2008. Survival was calculated from the date of diagnosis until 13 January 2009, when any remaining survivors were censored. During the study period, patients in the neoadjuvant arm with adenocarcinomas were treated with either neoadjuvant ECF (epirubicin (50 mg m^–2^), cisplatin (60 mg m^–2^) and continuous infusional 5-FU (200 mg m^–2^ per day)) or ECX (epirubicin (50 mg m^–2^), cisplatin (60 mg m^–2^) and capecetabine (625 mg m^–2^ p.o. b.d continuously)) chemotherapy up to three cycles before surgery. Patients with squamous cell carcinoma were treated with CF (cisplatin (80 mg m^–2^) and infusional 5-FU (1000 mg m^–2^ daily for 4 days)) (Allum *et al*, 2009) chemotherapy up to two cycles before surgery. The conduct of this study was approved by the ethics committee of Nottingham University Hospitals.

### Construction of TMA

Tissue microarrays were constructed as described previously (Kononen *et al*, 1998). In short, area-specialised histopathologists identified and marked formalin-fixed paraffin-embedded tissue blocks containing tumour tissue on haematoxylin and eosin-stained slides. The marked areas in these donor paraffin blocks were used to construct the TMA. Triplicate tissue cores with a diameter of 0.6 mm were taken from the marked areas and arrayed into a recipient paraffin block using a tissue puncher/arrayer (Beecher Instruments, Silver Spring, MD, USA) as previously described (Kononen *et al*, 1998). Sections of the tissue array block (5 *μ*m) were cut and placed on Fisherbrand Colorfrost/Plus microscope slides (Fisher Scientific, Pittsburgh, PA, USA) for immunohistochemical staining.

### Immunohistochemistry (IHC)

A standard streptavidin-biotin-peroxidase complex method was used. Negative controls were obtained by omitting the primary antibody in each case. The tissue slides were deparaffinised with xylene and then rehydrated through five decreasing concentrations of alcohol (100, 90, 70, 50 and 30%) for 2 min each. Endogenous peroxidise activity was blocked by incubation in a 1% hydrogen peroxide/methanol buffer. Antigen retrieval was carried out by microwave treatment of the slides in sodium citrate buffer (pH 6.0) for 10 min. The slides were rinsed in phosphate buffer solution (PBS) and incubated with blocking serum diluted in PBS to block nonspecific staining. For ERCC1 analysis the slides were incubated for 1 h with the primary anti-ERCC1 antibody (Santa Cruz Biotechnology, Inc., Santa Cruz, CA, USA) in a dilution of 1 : 50. For XPF analysis the slides were incubated for 1 h with the primary anti-XPF antibody (Abcam Plc, Cambridge, UK) in a dilution of 1 : 50. For FANCD2 analysis the slides were incubated for 1 h with the primary anti-FANCD2 antibody (Novus Biologicals Inc., Littleton, CO, USA) in a dilution of 1 : 200. For APE1 analysis the slides were incubated for 1 h with the primary anti-APE1 antibody (Novus Biologicals) in a dilution of 1 : 500. For p53 analysis the slides were incubated for 1 h with the primary anti-p53 antibody (Vector Labs, Burlingame, CA, USA) in a dilution of 1 : 50. All primary antibody dilutions were made in PBS. After washing with PBS, sections were incubated with the secondary antibody (Vector Labs) for 30 min followed by the avidin-biotin complex for a further 30 min. 3-3′ Diaminobenzidine tetrahydochloride was used as a chromogen. All sections were counterstained with Gill's haematoxylin.

### Evaluation of immune staining

The tumour cores were evaluated by specialist pathologist and oncologist together who were blinded to the clinico-pathological characteristics of patients. A consensus score was agreed for each core by the investigators.

Whole field inspection of the core was included in the assessment, and intensities of staining were grouped as follows: 0=no staining, 1=weak staining, 2=moderate staining and 3=strong staining. Nuclear staining was assessed separately for each core. Strong, moderate or weak nuclear staining was considered as positive staining. For p53 staining analysis, the level of protein accumulation was scored as 0 (no detectable immunostain), 1 (few nuclei), 2 (up to 10% nuclei), 3 (10–50% nuclei) and 4 (>50% nuclei) based on previously published literature (Shiao *et al*, 1998). Only stained malignant cells were included in the evaluation of staining. Not all cores within the TMA were suitable for IHC analyses because of small technical problems such as some cores were missing or lacked tumours. Only adenocarcinomas were included in the immunohistochemical analyses.

### Statistical analysis

Statistical analysis of data was performed using SPSS version 15.0 for Windows (SPSS Inc., Chicago, IL, USA). Univariate analysis of associations was determined using Pearson's *χ*^2^ test. Survival rates were calculated from the time of diagnosis until the end of the follow-up period and Kaplan–Meier curves were plotted. The statistical significance of differences between survival rates was determined using the log-rank test. Survival was censored if the patient was still alive. The *P-*values of <0.05 were identified as statistically significant.

## Results

### Patient demographics

There were two groups of patients: those who received at least one cycle of neoadjuvant chemotherapy (neoadjuvant group) and those who underwent primary surgery (primary group; Table 1). There were 103 patients in the neoadjuvant group, with a median age of 63 years and 81% were males. In this group, T3 tumours were the majority, making 62% of cases. The primary group had 142 cases, with a median age of 74 years, in which 74% were males and 53% had T3 tumours. In addition, 78% had received all the planned three cycles of neoadjuvant ECF/ECX chemotherapy (adenocarcinomas) and 96.4% had received all the planned two cycles of neoadjuvant CF chemotherapy (squamous cell carcinomas) in the neoadjuvant group. Of the patients who received all three cycles of ECF/ECX chemotherapy, 42% went on to receive the remaining three cycles of ECF/ECX chemotherapy. There was no significant difference between the primary surgery group and perioperative chemotherapy group (gastric/gastro-oesophageal junction) with regard to T stage (T2 (37 *vs* 33.3%) and T3 (48.4 *vs* 40%)) and N stage (N0 (27 *vs* 37.8%) and >N0 (73 *vs* 62.2%)). Only adenocarcinomas were included in the immunohistochemical and survival analyses in this study.

### TRG and survival

**Figure 1:**
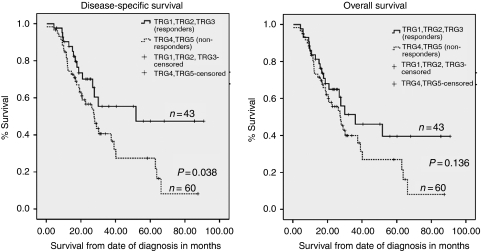
Kaplan–Meier curves representing the relationship between tumour regression grade (TRG) and disease-specific and overall survival in months from time of diagnosis in patients having received neoadjuvant chemotherapy.

Our previous study reported the utility of TRG analysis as a histopathological marker of response to neoadjuvant chemotherapy and tumour downstaging (Fareed *et al*, 2009). However, at the time of publication of that study long term follow-up clinical data were not available. In this study we show that patients who received neoadjuvant chemotherapy and achieved favourable tumour regression (TRG 1, 2 or 3) showed significantly better disease-specific survival compared with non-responders (Figure 1). The median disease-specific survival in TRG 1–3 (responders) was 51.7 *vs* 27.6 months in TRG 4 and 5 (non-responders; *P*=0.038). The median overall survival in TRG 1–3 (responders) was 36.1 *vs* 27.6 months in TRG4-5 (non-responders; *P*=0.136).

**Figure 2:**
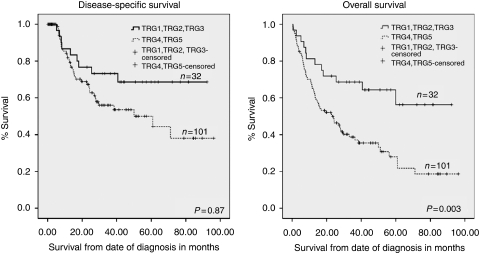
Kaplan–Meier curves representing the relationship between tumour regression grade (TRG) and disease-specific and overall survival in months from time of diagnosis in patients who received surgery only.

In the primary surgery group in which patients did not receive neoadjuvant chemotherapy, we found that patients whose tumours had spontaneous regression (TRG 1, 2 or 3) had a favourable mean overall survival of 61.8 months compared with 36.5 months in TRG 4and 5 group (*P*=0.003; Figure 2). Similar trend was also observed for disease-specific survival, although it did not reach statistical significance (mean survival 68.6 months (TRG 1–3) compared with 53.9 months (TRG 4 and 5); *P*=0.87; Figure 2).

### Immunohistochemical analyses and clinicopathological correlations

**Figure 3:**
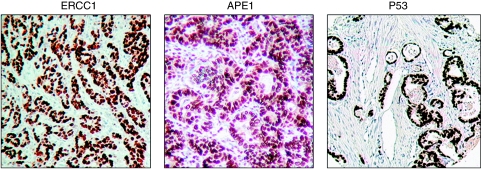
Microphotographs of ERCC1 (strongly positive), APE1 (strongly positive) and p53 (>50% nuclei staining) immunohistochemical staining showing nuclear expression in tissue microarray cores (magnification × 100).

**Figure 4:**
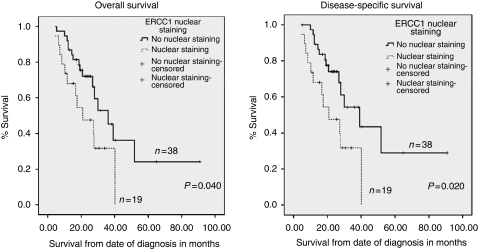
Kaplan–Meier curves representing the relationship between ERCC1 nuclear expression and disease-specific and overall survival in months from the time of diagnosis in the neoadjuvant group (*n*=57 patients).

*ERCC1* A total of 57 cores were suitable for analyses in the neoadjuvant group. Out of 57, 28 (49.2%) were ERCC1 positive and 29 (50.8%) were ERCC1 negative in the nucleus (Figure 3 and Table 2). Tumours that were ERCC1 positive showed no histopathological response to chemotherapy as evidenced by a TRG score of 4 or 5. This was statistically significant (*P*=0.006; Table 2). There were no significant associations between ERCC1 expression and other variables such as late T stage (T3 or T4), nodal involvement, vascular or perineural involvement. The median disease-specific survival in nuclear-positive ERCC1 patients was 20.9 *vs* 39.1 months in nuclear-negative patients (*P*=0.020). The median overall survival in nuclear-positive ERCC1 patients was 20.9 *vs* 36.1 months in nuclear-negative patients (*P*=0.040; Figure 4).

In the primary surgery group, 94 cores were suitable for analyses. ERCC1 positivity was frequently observed (69 out of 94 (73.4%)) but this did not correlate with any clinicopathological variables. ERCC1 nuclear expression was not associated with disease-specific (*P*=0.956) or overall survival (*P*=0.905).

*XPF* A total of 61 cores were suitable for analyses in the neoadjuvant group and 102 cores in primary surgery group. Nuclear XPF expression was frequently observed (neoadjuvant group 55 out of 61 (90.2%) and primary surgery group 101 out of 102 (99%)). There was no correlation with TRG and other clinicopathological variables.

*FANCD2* A total of 57 cores were suitable for analyses in the neoadjuvant group and 91 cores in the primary surgery group. Nuclear XPF expression was observed (neoadjuvant group 37 out of 57 (65%) and primary surgery group 59 out of 91 (65%)). There was no correlation with TRG and other clinicopathological variables.

**Figure 5:**
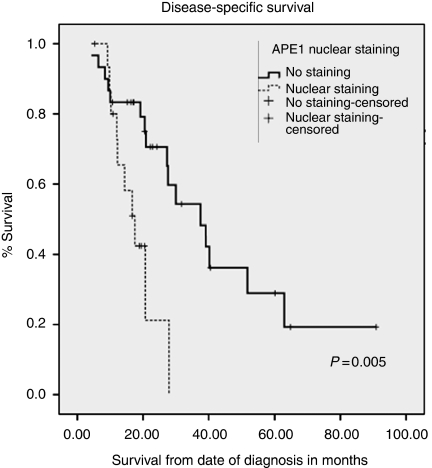
Kaplan–Meier curves representing the relationship between APE1 expression and disease-specific survival in the neoadjuvant group (*n*=46 patients).

*APE1* A total of 46 cores were suitable for analyses in the neoadjuvant group and 93 cores in primary surgery group. Nuclear APE1 expression was observed (neoadjuvant group, 28 out of 46 (60.9%) and primary surgery group, 63 out of 93 (67.7%); Figure 3 and Table 2). There was no correlation with TRG, T stage, N stage, vascular or perineural invasion. Interestingly, in the neoadjuvant group, median disease-specific survival in nuclear-positive APE1 patients was 17.5 *vs* 37.5 months in nuclear-negative patients. This was statistically significant (*P*=0.005; Figure 5).

**Figure 6:**
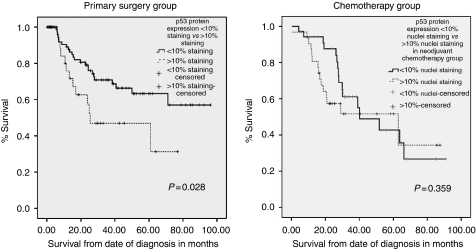
Kaplan–Meier curves representing the relationship between p53 nuclear expression and disease-specific survival in the primary surgery group.

*p53* A total of 66 cores were suitable for analyses in the neoadjuvant group and 122 cores in the primary surgery group. Nuclear p53 expression was frequently observed (neoadjuvant group, 35 out of 66 (53%) and primary surgery group, 39 out of 122 (32%); Figure 3 and Table 2). In the primary surgery group p53 positivity significantly correlated with nodal involvement (*P*=0.016) and perineural invasion (0.023). The mean disease-specific survival in patients expressing >10% nuclei p53 staining was 41.5 *vs* 67.2 months in those expressing <10% nuclei staining (*P*=0.028; Figure 6). The median overall survival in patients expressing >10% nuclei p53 staining was 26.7 *vs* 53.2 months in those expressing <10% nuclei staining (*P*<0.001). No significant association was observed in the neoadjuvant chemotherapy group (Figure 6).

## Discussion

The role of multimodality therapy in improving patient outcomes is generally accepted but remains controversial in gastro-oesophageal cancers. Current evidence suggests that preoperative treatment does not adversely affect surgical outcomes (Cunningham *et al*, 2006). However, only those patients who respond to preoperative therapy with tolerable toxicity will potentially benefit from this approach. The risk of delaying surgery in those patients who do not respond to chemotherapy may negatively influence clinical outcome. Moreover, the role of adjuvant chemotherapy in patients who have received neoadjuvant chemotherapy is uncertain, particularly in patients who show no evidence of tumour response to preoperative chemotherapy. Reliable evaluation of tumour response in the surgical resection specimens would be helpful in planning postoperative chemotherapy. In addition, predictive markers of response would be invaluable in individualising patient treatment as it would enable discrimination of those patients likely to respond to combination therapy from those likely to be non-responsive. In particular, for those patients who had achieved little or no response to preoperative chemotherapy, the use of alternative forms of adjuvant therapy could be considered to improve outcomes. Until the recent incorporation of neoadjuvant chemotherapy as a standard treatment option for operable gastro-oesophageal tumours (Cunningham *et al*, 2006), fit patients routinely received surgery in our centre. Therefore, the first set consisted of 142 gastric cancer cases not exposed to neoadjuvant chemotherapy. Biomarker investigations in this set may provide prognostic information in patients. With the incorporation of neoadjuvant chemotherapy since 2006, we also established a second TMA of 103 gastric/gastro-oesophageal cancer cases exposed to preoperative platinum-based chemotherapy. Biomarker investigations in this set may provide predictive information in patients.

In the previous study we provided the first evidence that TRG analysis correlate with tumour downstaging in gastro-oesophageal tumours (Fareed *et al*, 2009). In this study we have shown that lack of histopathological response to preoperative chemotherapy is associated with poor disease-specific survival (*P*=0.038). Our study suggests the potential need for alternative chemotherapy strategies in adjuvant setting in non-responders. However, larger studies would be required to confirm our findings and to allow design of clinical trials to address this clinical problem. In our previous study we reported on spontaneous regression of tumours not exposed to chemotherapy (Fareed *et al*, 2009). In this study we present the first evidence to suggest that spontaneous regression in tumour may be associated with better overall survival. The most extensive TRG in patients who had not received chemotherapy was in patients with predominantly mucosal disease, who had marked submucosal and muscularis propria fibrosis. In the absence of neoadjuvant chemotherapy, this could represent partial regression in tumours. Whether host immune factors such as lymphocytic infiltration could contribute to spontaneous regression is currently unknown and is an area of ongoing investigation. An alternative explanation is that this represents nonspecific scarring in an ulcer base due to a coexistent or preceding benign peptic ulcer with partial or complete mucosal tumour ‘healing’ overlying it (Fareed *et al*, 2009).

We then conducted an investigation of potential biomarkers that may have the ability to predict favourable/unfavourable TRG response ((TRG 1, 2 or 3) or (TRG 4 or 5) respectively). We focussed on key DNA repair and damage signalling factors, as the antitumour activity of platinum-based chemotherapy is largely dependent on the DNA repair capacity of cancer cells. In our study, 78% had received all the planned three cycles of neoadjuvant ECF/ECX chemotherapy for adenocarcinomas, and it is unlikely that this may have influenced the immunohistochemistry marker expression. We show that nuclear expression of ERCC1 is significantly associated with resistance to chemotherapy (TRG 4 or 5), implying that ERCC1 is a promising predictive marker. Moreover, we have also shown that nuclear ERCC1 expression correlates with poor disease-specific (*P*=0.005) and overall survival (*P*=0.005) in operable gastro-oesophageal tumours. However, in tumours not exposed to neoadjuvant chemotherapy, ERCC1 expression did not correlate with survival, implying that ERCC1 is likely to have predictive significance rather than prognostic significance in these tumours.

Elevated ERCC1 mRNA expression has previously been shown to predict resistance to chemotherapy in gastro-oesopahgeal tumours (Metzger *et al*, 1998; Warnecke-Eberz *et al*, 2004; Joshi *et al*, 2005). The ability of ERCC1 mRNA levels in predicting response has also been shown in lung (Lord *et al*, 2002; Simon *et al*, 2005), colorectal (Shirota *et al*, 2001), ovarian (Dabholkar *et al*, 1992, 1994) and bladder cancer (Bellmunt *et al*, 2007).

A recent study used immunohistochemistry to examine ERCC1 expression in the primary tumours of 64 patients with advanced gastric cancer treated with 5-FU/oxaliplatin chemotherapy. Patients without ERCC1 expression were more likely to respond to chemotherapy, and this was also associated with significantly longer median overall survival (Kwon *et al*, 2007). Our results are consistent with the above findings. In addition, we have provided the first evidence of ERCC1 protein expression in early-stage gastro-oesophageal tumours using immunohistochemistry. Moreover, our results are consistent with a recent study in lung cancer patients. The IALT Biology Study used immunohistochemical analysis to determine the expression of the ERCC1 protein in the operative lung cancer specimens. Among 761 tumours, ERCC1 expression was positive in 335 (44%) and negative in 426 (56%). A benefit from cisplatin-based adjuvant chemotherapy was associated with the absence of ERCC1 (Olaussen *et al*, 2006).

Nuclear expression of XPF, FANCD2, p53 and APE1 did not correlate with TRG response. However, nuclear expression of APE1 in the neoadjuvant group correlated with worse disease-specific (*P*=0.020) and overall survival (*P*=0.040). No significant correlations were observed in tumours not exposed to neoadjuvant chemotherapy. We have recently shown a similar correlation in ovarian cancer in which nuclear expression was associated with worse survival. In addition, APE1 expression was associated with a trend towards platinum resistance in patients (*P*=0.07; Al-Attar *et al*, 2010). Nuclear p53 correlated with disease-specific (*P*=0.028) and overall survival (*P*<0.001) in the primary surgery group but no significant associations were observed in the neoadjuvant group. This is in contrast to a study by Kamoshida *et al* (2007), who showed that p53 expression was correlated with resistance to chemotherapy in gastric cancer patients.

Our study is limited by the retrospective design and small numbers of tumours. We have provided evidence that TRG correlates with survival and ERCC1 nuclear expression is associated with resistance to chemotherapy as assessed by TRG and is also associated with poor disease-specific and overall survival. Larger studies are needed to validate this observation that is likely to have important clinical implications for patients receiving neoadjuvant chemotherapy for gastro-oesophageal cancer.

## Figures and Tables

**Table 1:** Patients’ demographics

	**Number (%) (neoadjuvant chemotherapy group)**	**Number (%) (primary surgery group)**
*Total number of patients*	103	142
*Median age*	63 years	74 years
		
*Sex*		
Male	83 (81%)	105 (73.9%)
Female	20 (19%)	37 (26%)
		
*T stage*		
T1	4 (3.8%)	14 (9.8%)
T2	24 (23.6)	48 (33.8%)
T3	64 (62%)	75 (52.8%)
T4	9 (8.6%)	5 (3.5%)
TX	2 (2%)	
		
*N stage*		
N0	29 (28%)	33 (23.2%)
⩾N1	74 (72%)	109 (76.8%)
		
*M stage*		
M0	103 (100%)	140 (98.5%)
M1	—	2 (1.4%)
		
*Tumour type*		
Adenocarcinoma	88 (85.4%)	142 (100%)
Squamous cell carcinoma	13 (12.6%)	—
Adenosquamous	2 (1.9%)	—
		
*Site of tumour*		
Gastric	20 (19.4%)	142 (100%)
GOJ	47 (45.6%)	—
Lower third of oesophagus	36 (35%)	—
		
*Surgery*		
Total gastrectomy	22	70
Partial gastrectomy	5	52
Oesophagectomy/oesophago- gastrectomy	76	20
		
*TRG response after chemotherapy*		
1, 2 and 3	43 (41.7%)	—
4 and 5	60 (58.3%)	—
		
*Survival status*		
Alive	47 (46%)	54 (38%)
Dead	56 (54%)	87 (62%)

Abbreviations: GOJ=gastro-oesophageal junction; TRG=tumour regression grade.

**Table 2:** Nuclear expression and correlation with lack of tumour response (TRG 4 or 5) in the neoadjuvant chemotherapy group

	**Positive (%)**	**Negative (%)**	**Lack of tumour response to chemotherapy**
ERCC1	28/57 (49.1%)	29/57 (50.8%)	0.006^a^
XPF	55/61 (90.2%)	6/61 (9.8%)	0.498
FANCD2	37/57 (65%)	20/57 (35%)	1.0
APE1	28/46 (60.9%)	18/46 (39.1%)	0.295
TP53	35/66 (53%)	31/66 (47%)	0.706

Abbreviation: TRG=tumour regression grade.

a Positive nuclear staining significantly correlated with lack of tumour response (i.e., TRG 4 or 5).

## References

[bib1] Al-Attar A, Gossage L, Fareed K, Shehata M, Abbotts R, Zaitoun A, Soomro I, Lobo DN, Parson SL, Chan S, Madhusudan S (2010) Human apurinic/apyrimidinic endonuclease (APE1) is a potential drug target in melanoma, ovarian, gastric and pancreatico-biliary cancers. Br J Cancer 102: 704–709 20087352 10.1038/sj.bjc.6605541 PMC2837561

[bib2] Allum WH, Stenning SP, Bancewicz J, Clark PI, Langley RE (2009) Long-term results of a randomized trial of surgery with or without preoperative chemotherapy in esophageal cancer. J Clin Oncol 27: 5062–5067 19770374 10.1200/JCO.2009.22.2083

[bib3] Bartz SR, Zhang Z, Burchard J, Imakura M, Martin M, Palmieri A, Needham R, Guo J, Gordon M, Chung N, Warrener P, Jackson AL, Carleton M, Oatley M, Locco L, Santini F, Smith T, Kunapuli P, Ferrer M, Strulovici B, Friend SH, Linsley PS (2006) Small interfering RNA screens reveal enhanced cisplatin cytotoxicity in tumor cells having both BRCA network and TP53 disruptions. Mol Cell Biol 26: 9377–9386 17000754 10.1128/MCB.01229-06 PMC1698535

[bib4] Bellmunt J, Paz-Ares L, Cuello M, Cecere F, Albiol S, Guillem V, Gallardo E, Carles J, Mendez P, de la Cruz J, Taron M, Rosell R, Baselga J (2007) Gene expression of ERCC1 as a novel prognostic marker in advanced bladder cancer patients receiving cisplatin-based chemotherapy. Ann Oncol 18: 522–528 17229776 10.1093/annonc/mdl435

[bib5] Cepeda V, Fuertes MA, Castilla J, Alonso C, Quevedo C, Perez JM (2007) Biochemical mechanisms of cisplatin cytotoxicity. Anticancer Agents Med Chem 7: 3–18 17266502 10.2174/187152007779314044

[bib6] Chen Q, Van der Sluis PC, Boulware D, Hazlehurst LA, Dalton WS (2005) The FA/BRCA pathway is involved in melphalan-induced DNA interstrand cross-link repair and accounts for melphalan resistance in multiple myeloma cells. Blood 106: 698–705 15802532 10.1182/blood-2004-11-4286 PMC1895179

[bib7] Cunningham D, Allum WH, Stenning SP, Thompson JN, Van de Velde CJ, Nicolson M, Scarffe JH, Lofts FJ, Falk SJ, Iveson TJ, Smith DB, Langley RE, Verma M, Weeden S, Chua YJ, Participants MT (2006) Perioperative chemotherapy versus surgery alone for resectable gastroesophageal cancer. N Engl J Med 355: 11–20 16822992 10.1056/NEJMoa055531

[bib8] Cunningham D, Starling N, Rao S, Iveson T, Nicolson M, Coxon F, Middleton G, Daniel F, Oates J, Norman AR (2008) Capecitabine and oxaliplatin for advanced esophagogastric cancer. N Engl J Med 358: 36–46 18172173 10.1056/NEJMoa073149

[bib9] D’Andrea AD, Grompe M (2003) The Fanconi anaemia/BRCA pathway. Nat Rev Cancer 3: 23–34 12509764 10.1038/nrc970

[bib10] Dabholkar M, Bostick-Bruton F, Weber C, Bohr VA, Egwuagu C, Reed E (1992) ERCC1 and ERCC2 expression in malignant tissues from ovarian cancer patients. J Natl Cancer Inst 84: 1512–1517 1433335 10.1093/jnci/84.19.1512

[bib11] Dabholkar M, Vionnet J, Bostick-Bruton F, Yu JJ, Reed E (1994) Messenger RNA levels of XPAC and ERCC1 in ovarian cancer tissue correlate with response to platinum-based chemotherapy. J Clin Invest 94: 703–708 8040325 10.1172/JCI117388 PMC296149

[bib12] Demple B, Sung JS (2005) Molecular and biological roles of Ape1 protein in mammalian base excision repair. DNA Repair 4: 1442–1449 16199212 10.1016/j.dnarep.2005.09.004

[bib13] Fareed KR, Ilyas M, Kaye PV, Soomro IN, Lobo DN, Parsons SL, Madhusudan S (2009) Tumour regression grade (TRG) analyses in patients with resectable gastro-oesophageal adenocarcinomas treated with platinum-based neoadjuvant chemotherapy. Histopathology 55: 399–406 19817890 10.1111/j.1365-2559.2009.03404.x

[bib14] Gillet LC, Scharer OD (2006) Molecular mechanisms of mammalian global genome nucleotide excision repair. Chem Rev 106: 253–276 16464005 10.1021/cr040483f

[bib15] Gossage L, Madhusudan S (2007) Current status of excision repair cross complementing-group 1 (ERCC1) in cancer. Cancer Treat Rev 33: 565–577 17707593 10.1016/j.ctrv.2007.07.001

[bib16] Joshi MB, Shirota Y, Danenberg KD, Conlon DH, Salonga DS, Herndon II JE, Danenberg PV, Harpole Jr DH (2005) High gene expression of TS1, GSTP1, and ERCC1 are risk factors for survival in patients treated with trimodality therapy for esophageal cancer. Clin Cancer Res 11: 2215–2221 15788669 10.1158/1078-0432.CCR-04-1387

[bib17] Kamoshida S, Suzuki M, Shimomura R, Sakurai Y, Komori Y, Uyama I, Tsutsumi Y (2007) Immunostaining of thymidylate synthase and p53 for predicting chemoresistance to S-1/cisplatin in gastric cancer. Br J Cancer 96: 277–283 17211470 10.1038/sj.bjc.6603546 PMC2360001

[bib18] Kononen J, Bubendorf L, Kallioniemi A, Barlund M, Schraml P, Leighton S, Torhorst J, Mihatsch MJ, Sauter G, Kallioniemi OP (1998) Tissue microarrays for high-throughput molecular profiling of tumor specimens. Nat Med 4: 844–847 9662379 10.1038/nm0798-844

[bib19] Kwon HC, Roh M, Oh S, Kim SH, Kim M, Kim JS, Kim HJ (2007) Prognostic value of expression of ERCC1, thymidylate synthase, and glutathione S-transferase P1 for 5-fluorouracil/oxaliplatin chemotherapy in advanced gastric cancer. Ann Oncol 18: 504–509 17322540 10.1093/annonc/mdl430

[bib20] Levitus M, Joenje H, de Winter JP (2006) The Fanconi anemia pathway of genomic maintenance. Cell Oncol 28: 3–29 16675878 10.1155/2006/974975 PMC4617492

[bib21] Lord RV, Brabender J, Gandara D, Alberola V, Camps C, Domine M, Cardenal F, Sanchez JM, Gumerlock PH, Taron M, Sanchez JJ, Danenberg KD, Danenberg PV, Rosell R (2002) Low ERCC1 expression correlates with prolonged survival after cisplatin plus gemcitabine chemotherapy in non-small cell lung cancer. Clin Cancer Res 8: 2286–2291 12114432

[bib22] Madhusudan S, Hickson ID (2005) DNA repair inhibition: a selective tumour targeting strategy. Trends Mol Med 11: 503–511 16214418 10.1016/j.molmed.2005.09.004

[bib23] Madhusudan S, Middleton MR (2005) The emerging role of DNA repair proteins as predictive, prognostic and therapeutic targets in cancer. Cancer Treat Rev 31: 603–617 16298073 10.1016/j.ctrv.2005.09.006

[bib24] Mandard AM, Dalibard F, Mandard JC, Marnay J, Henry-Amar M, Petiot JF, Roussel A, Jacob JH, Segol P, Samama G (1994) Pathologic assessment of tumor regression after preoperative chemoradiotherapy of esophageal carcinoma. Clinicopathologic correlations. Cancer 73: 2680–2686 8194005 10.1002/1097-0142(19940601)73:11<2680::aid-cncr2820731105>3.0.co;2-c

[bib25] Medical Research Council Oesophageal Cancer Working Group (2002) Surgical resection with or without preoperative chemotherapy in oesophageal cancer: a randomised controlled trial. Lancet 359: 1727–1733 12049861 10.1016/S0140-6736(02)08651-8

[bib26] Metzger R, Leichman CG, Danenberg KD, Danenberg PV, Lenz HJ, Hayashi K, Groshen S, Salonga D, Cohen H, Laine L, Crookes P, Silberman H, Baranda J, Konda B, Leichman L (1998) ERCC1 mRNA levels complement thymidylate synthase mRNA levels in predicting response and survival for gastric cancer patients receiving combination cisplatin and fluorouracil chemotherapy. J Clin Oncol 16: 309–316 9440758 10.1200/JCO.1998.16.1.309

[bib27] Mirchandani KD, D’Andrea AD (2006) The Fanconi anemia/BRCA pathway: a coordinator of cross-link repair. Exp Cell Res 312: 2647–2653 16859679 10.1016/j.yexcr.2006.06.014

[bib28] Olaussen KA, Dunant A, Fouret P, Brambilla E, Andre F, Haddad V, Taranchon E, Filipits M, Pirker R, Popper HH, Stahel R, Sabatier L, Pignon JP, Tursz T, Le Chevalier T, Soria JC (2006) DNA repair by ERCC1 in non-small-cell lung cancer and cisplatin-based adjuvant chemotherapy. N Engl J Med 355: 983–991 16957145 10.1056/NEJMoa060570

[bib29] Rabik CA, Dolan ME (2007) Molecular mechanisms of resistance and toxicity associated with platinating agents. Cancer Treat Rev 33: 9–23 17084534 10.1016/j.ctrv.2006.09.006 PMC1855222

[bib30] Reardon JT, Sancar A (2005) Nucleotide excision repair. Prog Nucleic Acid Res Mol Biol 79: 183–235 16096029 10.1016/S0079-6603(04)79004-2

[bib31] Shiao YH, Palli D, Buzard GS, Caporaso NE, Amorosi A, Saieva C, Fraumeni Jr JF, Anderson LM, Rice JM (1998) Implications of p53 mutation spectrum for cancer etiology in gastric cancers of various histologic types from a high-risk area of central Italy. Carcinogenesis 19: 2145–2149 9886570 10.1093/carcin/19.12.2145

[bib32] Shirota Y, Stoehlmacher J, Brabender J, Xiong YP, Uetake H, Danenberg KD, Groshen S, Tsao-Wei DD, Danenberg PV, Lenz HJ (2001) ERCC1 and thymidylate synthase mRNA levels predict survival for colorectal cancer patients receiving combination oxaliplatin and fluorouracil chemotherapy. J Clin Oncol 19: 4298–4304 11731512 10.1200/JCO.2001.19.23.4298

[bib33] Siddik ZH (2003) Cisplatin: mode of cytotoxic action and molecular basis of resistance. Oncogene 22: 7265–7279 14576837 10.1038/sj.onc.1206933

[bib34] Simon GR, Sharma S, Cantor A, Smith P, Bepler G (2005) ERCC1 expression is a predictor of survival in resected patients with non-small cell lung cancer. Chest 127: 978–983 15764785 10.1378/chest.127.3.978

[bib35] van der Heijden MS, Brody JR, Dezentje DA, Gallmeier E, Cunningham SC, Swartz MJ, DeMarzo AM, Offerhaus GJ, Isacoff WH, Hruban RH, Kern SE (2005) *In vivo* therapeutic responses contingent on Fanconi anemia/BRCA2 status of the tumor. Clin Cancer Res 11: 7508–7515 16243825 10.1158/1078-0432.CCR-05-1048

[bib36] Wang D, Xiang DB, Yang XQ, Chen LS, Li MX, Zhong ZY, Zhang YS (2009) APE1 overexpression is associated with cisplatin resistance in non-small cell lung cancer and targeted inhibition of APE1 enhances the activity of cisplatin in A549 cells. Lung Cancer 66: 298–304 19324449 10.1016/j.lungcan.2009.02.019

[bib37] Wang W (2007) Emergence of a DNA-damage response network consisting of Fanconi anaemia and BRCA proteins. Nat Rev Genet 8: 735–748 17768402 10.1038/nrg2159

[bib38] Wang Y, Wiltshire T, Senft J, Wenger SL, Reed E, Wang W (2006) Fanconi anemia D2 protein confers chemoresistance in response to the anticancer agent, irofulven. Mol Cancer Ther 5: 3153–3161 17172419 10.1158/1535-7163.MCT-06-0427

[bib39] Warnecke-Eberz U, Metzger R, Miyazono F, Baldus SE, Neiss S, Brabender J, Schaefer H, Doerfler W, Bollschweiler E, Dienes HP, Mueller RP, Danenberg PV, Hoelscher AH, Schneider PM (2004) High specificity of quantitative excision repair cross-complementing 1 messenger RNA expression for prediction of minor histopathological response to neoadjuvant radiochemotherapy in esophageal cancer. Clin Cancer Res 10: 3794–3799 15173087 10.1158/1078-0432.CCR-03-0079

[bib40] Zhang J, Wang X, Lin CJ, Couch FJ, Fei P (2006) Altered expression of FANCL confers mitomycin C sensitivity in Calu-6 lung cancer cells. Cancer Biol Ther 5: 1632–1636 17106252 10.4161/cbt.5.12.3351

[bib41] Zhang Y, Wang J, Xiang D, Wang D, Xin X (2009) Alterations in the expression of the apurinic/apyrimidinic endonuclease-1/redox factor-1 (APE1/Ref-1) in human ovarian cancer and indentification of the therapeutic potential of APE1/Ref-1 inhibitor. Int J Oncol 35: 1069–1079 19787261

[bib42] Zorbas H, Keppler BK (2005) Cisplatin damage: are DNA repair proteins saviors or traitors to the cell? Chembiochem 6: 1157–1166 15934047 10.1002/cbic.200400427

